# Dynamics of plasma membrane surface related to the release of extracellular vesicles by mesenchymal stem cells in culture

**DOI:** 10.1038/s41598-017-07265-x

**Published:** 2017-07-28

**Authors:** Santiago Casado, Maria del Val Toledo Lobo, Carlos Luis Paíno

**Affiliations:** 1 Instituto Madrileño de Estudios Avanzados (IMDEA Nanoscience), Ciudad Universitaria de Cantoblanco, Madrid, Spain; 2 Departamento de Biomedicina y Biotecnología, IRYCIS, Universidad de Alcalá, Alcala de Henares, Spain; 3 Servicio de Neurobiología-Investigación, Hospital Universitario Ramón y Cajal, IRYCIS, Madrid, Spain

## Abstract

Extracellular vesicles (exosomes and shedding vesicles) released by mesenchymal stem cells (MSCs) are regarded as a storable, cell-free alternative with comparable therapeutic potential to their parent cells. Shedding vesicles originate as bulges on the cell surface but little is known about their turnover or how their formation can be stimulated. We have used atomic force microscopy (AFM) to follow the formation dynamics of bulges in living adipose tissue-derived MSCs. AFM images showed that, in general, MSCs present hundreds of nanosized protrusions on their surface with life spans of 10–20 min. Scanning electron microscopy confirmed those images and showed that bulges are also formed on filamentous processes. Extracellular vesicles deposited on the culture surface have comparable sizes to those of bulges showing up on the cell surface. The amount of protrusions on cells treated with progesterone or PDGF-BB, two treatments that stimulate the secretion of extracellular vesicles in MSCs, was evaluated by AFM. Measurements of the cross-area at 50 nm over the cell surface provided estimates of the amount of protrusions and showed that these values increased with the stimulating treatments. Our study suggests that shedding vesicles constitute a large population of the extracellular vesicle pool.

## Introduction

Extracellular vesicles (EVs) consist of membrane-enclosed globules packaging different types of biomolecules (proteins, nucleic acids, lipids and sugars)^1^ that can be transferred to near or distant cells through fluids and extracellular space. According to their mechanisms of formation and release, EVs can be classified into 3 main types: (A) Exosomes (40–120 nm) originating from the endolysosomal pathway and packaged in multivesicular bodies (MVB) that open to the extracellular space after fusing with the plasma membrane (B) Microvesicles or shedding vesicles (50–1000 nm) which develop as membrane protrusions, eventually giving rise to bulges that detach. (C) Apoptotic bodies, which are large vesicles (>500 nm) resulting from blebbing of apoptotic cell membranes^2^. Exosomes and microvesicles are actively generated by all healthy cells.

EVs play important roles in cell communication between close or distant target cells. Their physiological role includes immune modulation, intercellular signalling and tissue repair^3–5^ but they are also being tried as delivering vehicles of therapeutic molecules^6–8^. On the other hand, they also play roles in pathological processes like cancer metastasis^9, 10^ or the spread of pathogens^11–13^.

Adipose tissue-derived stromal cells (ADSCs) in culture are a type of mesenchymal stem cell (MSC)^14^ with great therapeutic potential, not only for their capability of deriving cells for cartilage and bone tissue remodelling or engineering^15–17^ but also because they show immunomodulatory, anti-inflammatory and neuroprotective properties^18–23^. Importantly, the therapeutic properties of MSCs can be directly driven by their EVs^24–26^. Because of that, it becomes relevant to know how EVs are secreted by ADSCs and how different drugs or conditions affect such secretion.

Few reports have studied microscopically the dynamics of EV formation and release. Given the small size of EVs, falling below the resolving power of optical instruments, most of the previous morphological studies have used electron microscopy, which produces a still picture of fixed cells in vacuum. Live-cell microscopy would allow tracking individual elements in the plasma membrane of a given cell. Superresolution fluorescent microscopy techniques for living cells may reach nanoscale spatial precision but, to date, have poor temporal resolution or entail limitations^27, 28^.

Atomic force microscopy (AFM) provides a tool for studying the surface of both fixed and living cells^29^ with a resolution power well beyond that of optic microscopy. For the study of nanometric-sized extracellular vesicles, AFM is thus a useful, though underutilized, instrument. Furthermore, AFM allows quantifying some characteristics of the studied surface, like height or stiffness, as well as to mechanically interact with it. In the present report, we have used AFM topographic imaging to study the dynamic of protuberances and pits on the surface of living ADSCs as well as to further analyse them in fixed cells. We propose that most of those protuberances will be shed as microvesicles and that pits might be the image of MVB fusing to the plasma membrane to release exosomes. To support that proposal, scanning electron microscopy (SEM) images will be compared with those of AFM, and drugs that increase the release of extracellular vesicles by ADSCs will be tested to check if AFM measurements of membrane protrusions parallel such increase.

## Results

**Figure 1:**
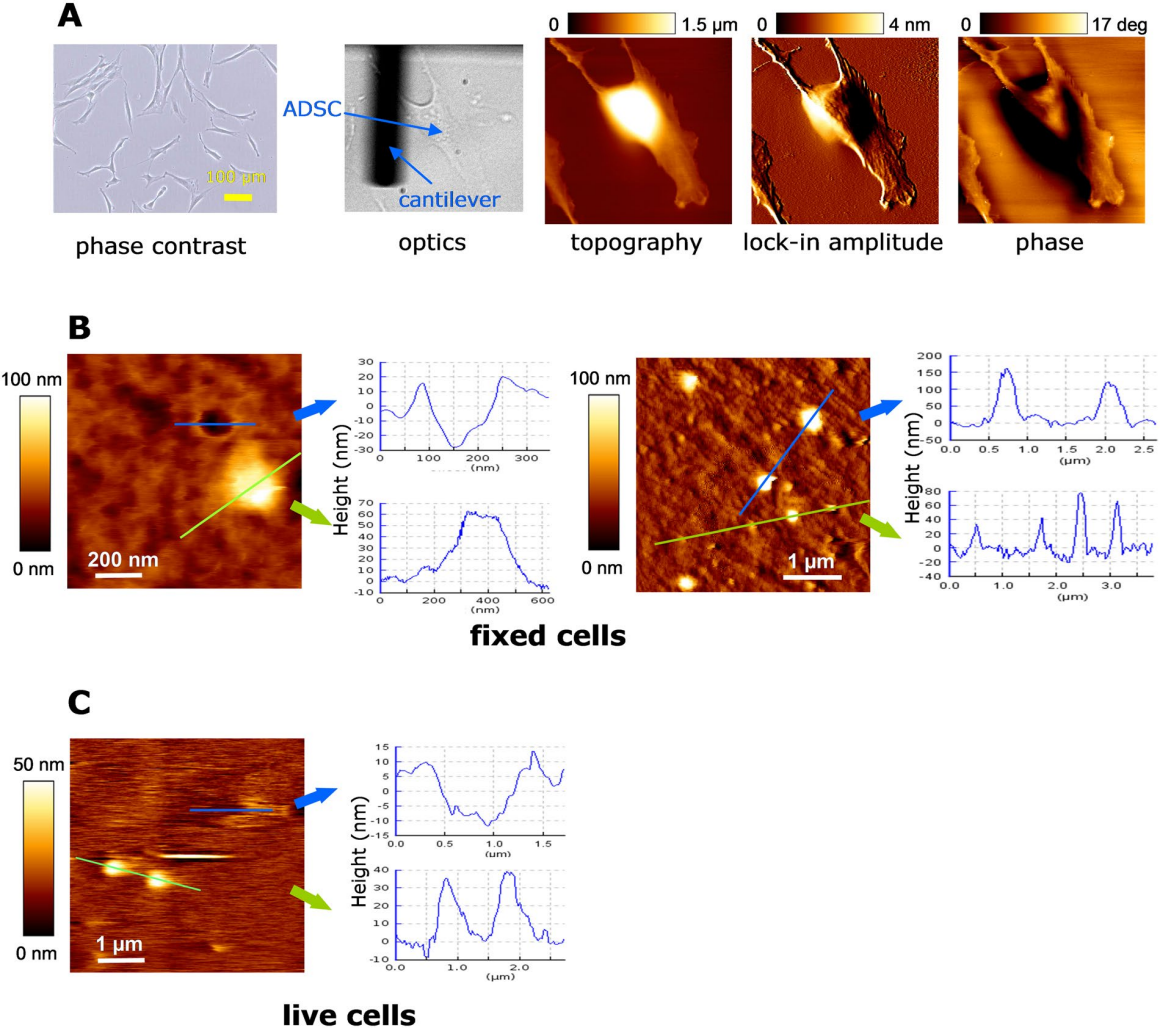
Morphology of protrusions and pits on ADSC membranes by AFM. (**A**) Characterization of cultured ADSCs under optic phase contrast, under direct light at the AFM set-up and under different AFM analyses (topography, lock-in amplitude and phase). (**B**) AFM measurements of fixed cells in two 10 × 10 µm fields. Medium sized (height: 100–200 nm) and small sized (height 20–100 nm) protrusions bulge on the surface. Also, circular, crater-like depressions with raised rims are present (measurements of the actual depth of pits is limited by probe tip constraints). (**C**) In living cell preparations at 37 °C, similar buddings and depressions were observed. We hypothesize that bulges are the origin of shedding vesicles and depressions are the surface appearance of membrane-fused MVBs for exosomes release.

The ADSC surface, as shown by AFM, presented numerous protruding bulges of various sizes, as well as some pits (Fig. 1). The height of protrusions could be measured with precision, most of them ranging from 20 nm to 300 nm. The width of protrusions was not considered a precise measurement due to geometrical constraints of the probe tip.

**Figure 2:**
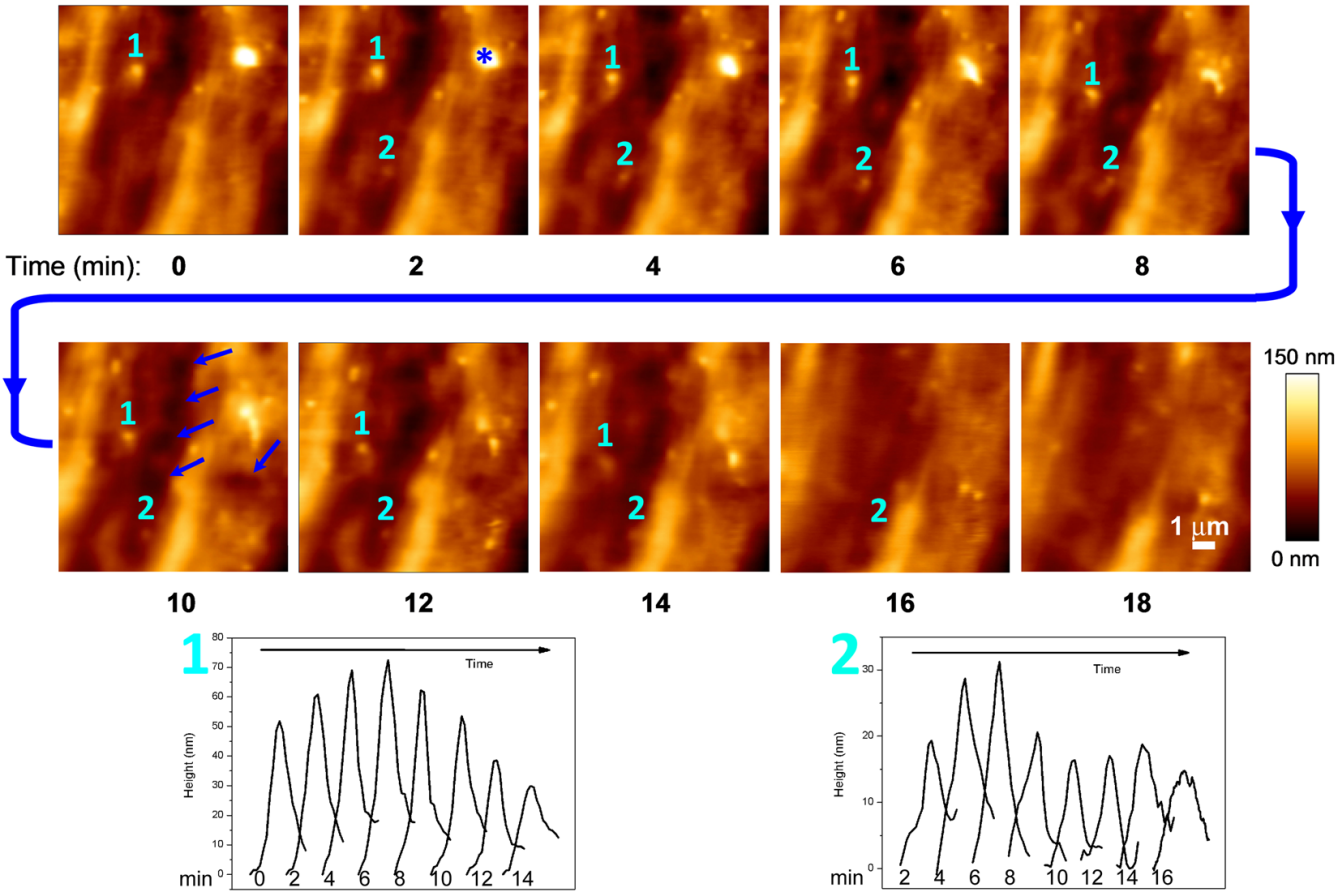
Sequence of AFM measurements in living cells. The topography of a 10 × 10 μm area on the cell surface of one cell is depicted. Lapse to record consecutive images at 128 × 128 pixels: 2 minutes (line rate around 1 Hz). The outer surface of ADSC membranes is highly dynamic and show protrusions that bulge, move and disappear within minutes. Below, the graphical evolution of two of these protrusions is depicted: #1 is a protrusion already present at time 0 that moves more than 2 µm before disappearing by min 16; #2 is a protrusion that emerges by min 2, grows and shows a sudden decrease by min 8, that can be interpreted as a partial shedding, while a smaller portion remains and disappears by min 18. Asterisk (*) marks a medium sized protrusion that divides into several smaller ones. Short-lived membrane depressions are also observed. Some of these depressions are of large diameter (arrowheads, sixth frame), while most of them are of small size. However, these small-sized depressions cannot be unambiguously identified as pits.

**Figure 3:**
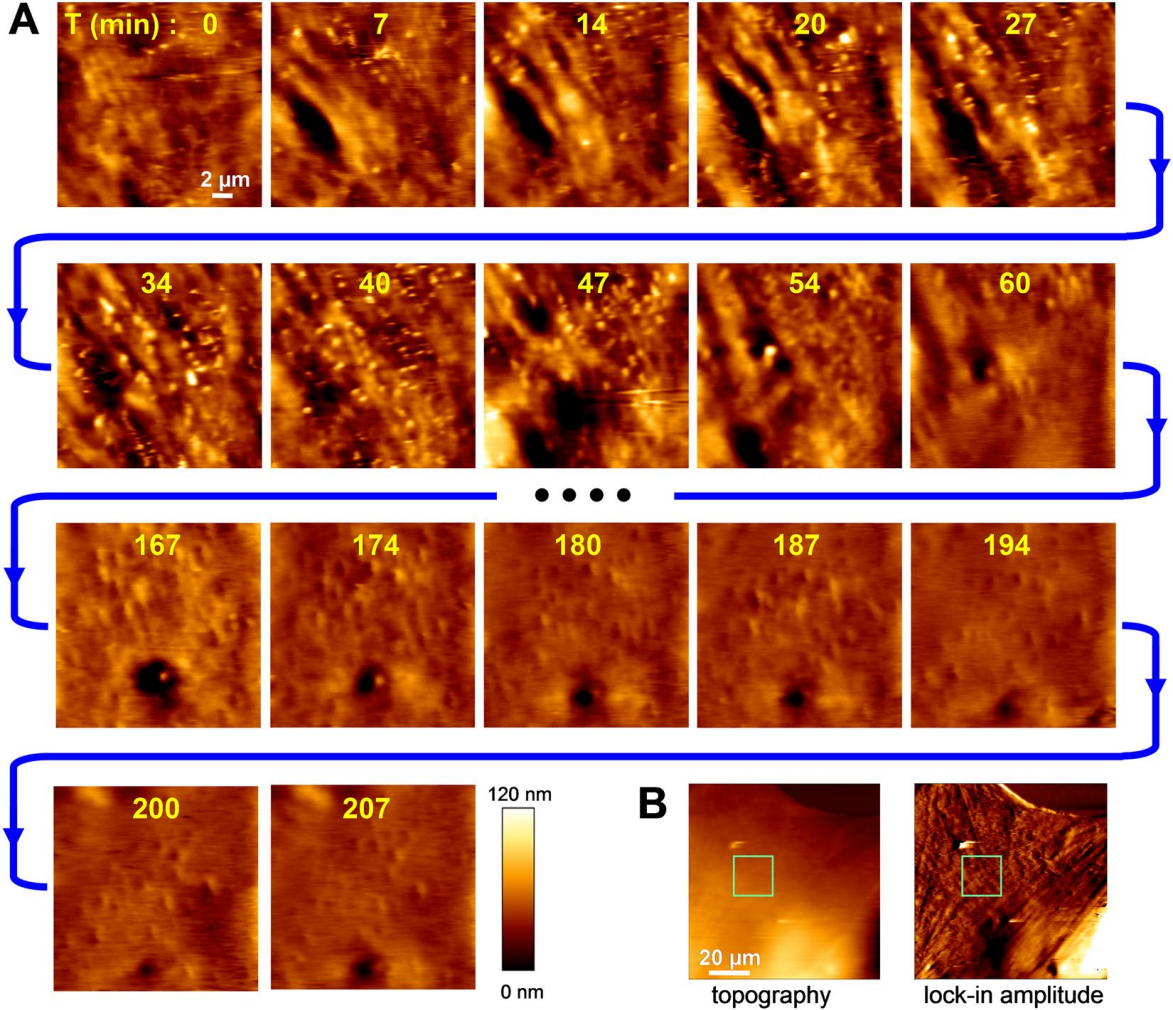
Long-lasting AFM measurements in living cells showing drastic changes in the cell surface morphology. (**A**) Sequence of images of cell surface topography (20 × 20 μm area, 256 × 256 pixels, line rate around 0.6 Hz), recorded every 7 minutes for 207 min. Recordings were started 3 h after progesterone addition. Images between min 60 and min 167 are not depicted here. The images show the turnover of protrusions for the initial 47 min. Afterwards, a drastic transition of the topography is observed. Besides the action of progesterone on ADSCs, cell movements and/or mechanical stimulation by the probe may be involved in such transition. (**B**) Images of topography and lock-in amplitude showing the area that was analysed (green square).

In living cells, bulges of all sizes showed a lifespan of 10–20 min in most cases (Figs 2 and 3), although few examples of longer or shorter-lived bulges were also observed. Large protrusions did not appear to grow from smaller ones, but they bud from the membrane already as wide bumps. Similarly, smaller protrusions appear and disappear at their final size. On occasions, after the disappearance of a protrusion, the surface remained elevated at that place for few minutes. We are not sure as if that represents remains of the bulge after shedding of the vesicle, if they consist in reabsorbed protrusions or even if they consist in EVs that are fusing with a target MSC to transfer their contents. Short displacements of protrusions (1–2 µm) in relation to their neighbours were also observed.

**Figure 4:**
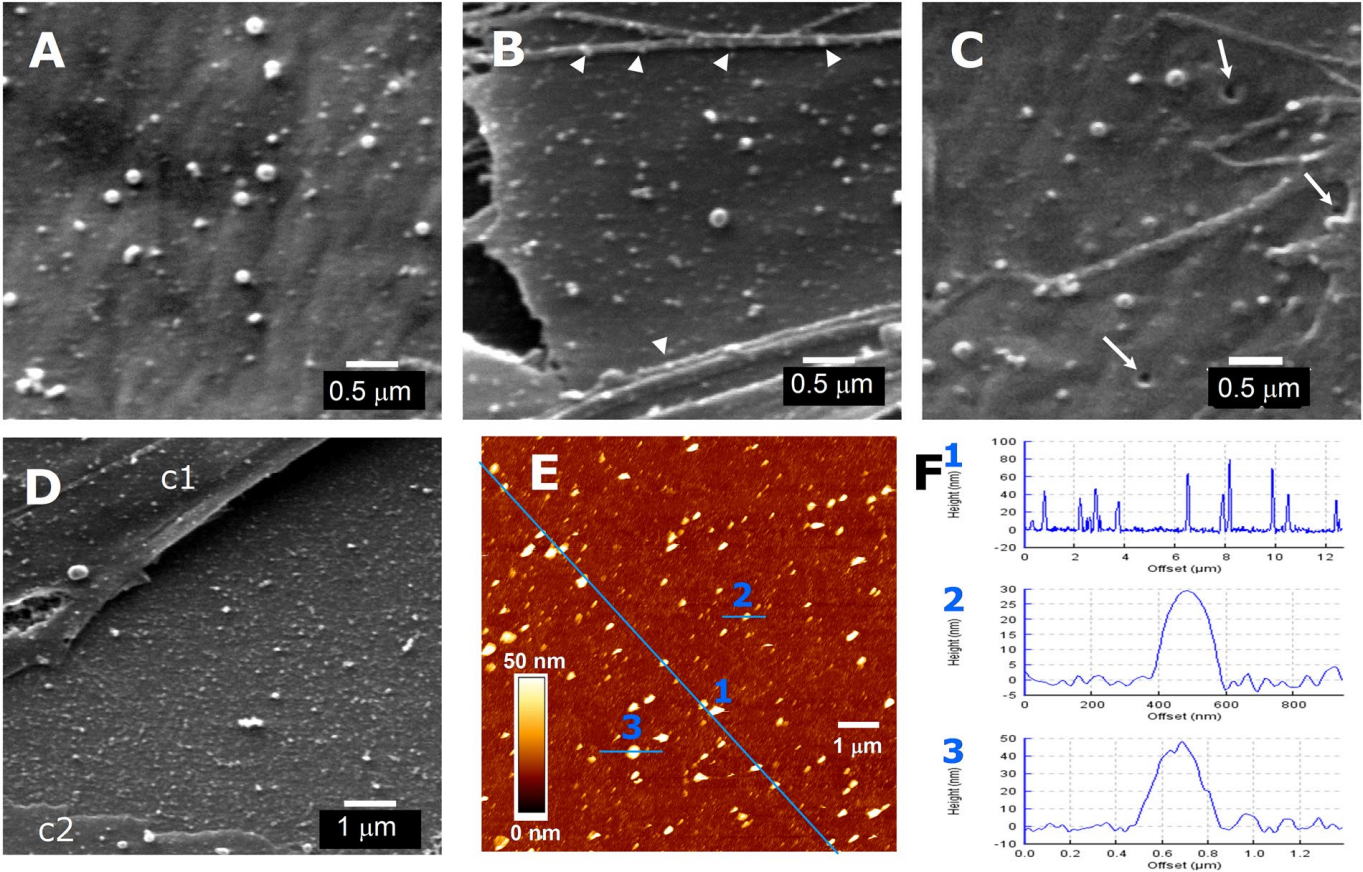
Scanning electron microscopy (SEM) images of protrusions and pits on ADSC surface and of EVs released by these cells. (**A**,**B**) Fields of ADSC surface showing protrusions of various sizes in control conditions (**A**) or 1 h after addition of 0.5 mM progesterone (**B**). Arrowheads point to some protrusions on filamentous processes. (**C**) Pits (arrows) were occasionally observed at the cell membrane. (**D**) Dense deposit of EVs on the coverslip surface between 2 cells (c1 and c2). (**E**) AFM image of ADSC-released EVs attached to poly-L-ornithine-treated coverslip. (**F**) Height profile samples of EVs attached to the coverslip area depicted in E, along the traces indicated in blue as 1, 2 and 3, respectively. Scale for A, B and C: 0.5 µm; for D and E: 1 µm. For F: abscise, trace length in µm (F2, in nm); ordinates, height in nm.

SEM images showed the same characteristics as AFM measurements in relation to the size and number of protruding bulges as well as of the less frequent pits (Fig. 4). Tenths to several hundred spherical or elongated elements, ranging from less than 100 nm to one micron in diameter, were observed by SEM on every ADSC surface. Many of them are clearly stemming from the membrane, which suggests that they are the possible origin of shedding vesicles. Some others might correspond to EVs released by neighbour cells that have attached to the membrane and, presumably, would be endocytosed and internalized^30^, although no image suggestive of phagocytic processes could be identified in our studies. In addition to the protrusions observed by AFM on the soma, large numbers of bulges of various sizes are shown by SEM on filamentous processes (Fig. 4B).

Less often, pits in the membrane were visible, both with AFM and with SEM. In living cells, pits measured by AFM had a radius of up to 2 µm (Figs 2 and 3) while those that were measured in fixed cells, either by AFM or by SEM never exceeded the 200 nm. Smaller pits could not be unambiguously identified by AFM but were observed in SEM images (Fig. 4C). By AFM, a crater-like morphology, i. e. a hole surrounded by raised edges, was typical of these pits (Fig. 1B). By SEM, raised edges were less evident. In living cells, largest pits could be observed to persist from few minutes for more than 1 h.

Additionally, deposition of secreted EVs on the culture surface was observed both with SEM and AFM. The intercellular spaces between cultured cells were crowded by vesicles of similar sizes as the protrusions that were observed on the neighbouring cells (Fig. 4D). We performed some experiments to estimate the amount of EVs that could be laid on a surface by a similar area of cultured cells. Coverslips cultured with ADSCs were placed upside-down lying on 1 mm-thick silicone rings over poly-L-ornithine coated coverslips, so that EVs released by the cells could sediment and attach to the empty surfaces underneath. After 24 h, the extracellular vesicle-deposited coverslips were fixed and AFM was performed. It could be observed that, similarly to what was shown by SEM, coverslip surfaces were densely laid with vesicles of different sizes (Fig. 4E). Attached EVs were flattened due to the adhesiveness of poly-L-ornithine coating, so their height was shorter (10–200 nm) than the corresponding to the diameter of the vesicles suspended in medium (Fig. 4F).

**Figure 5:**
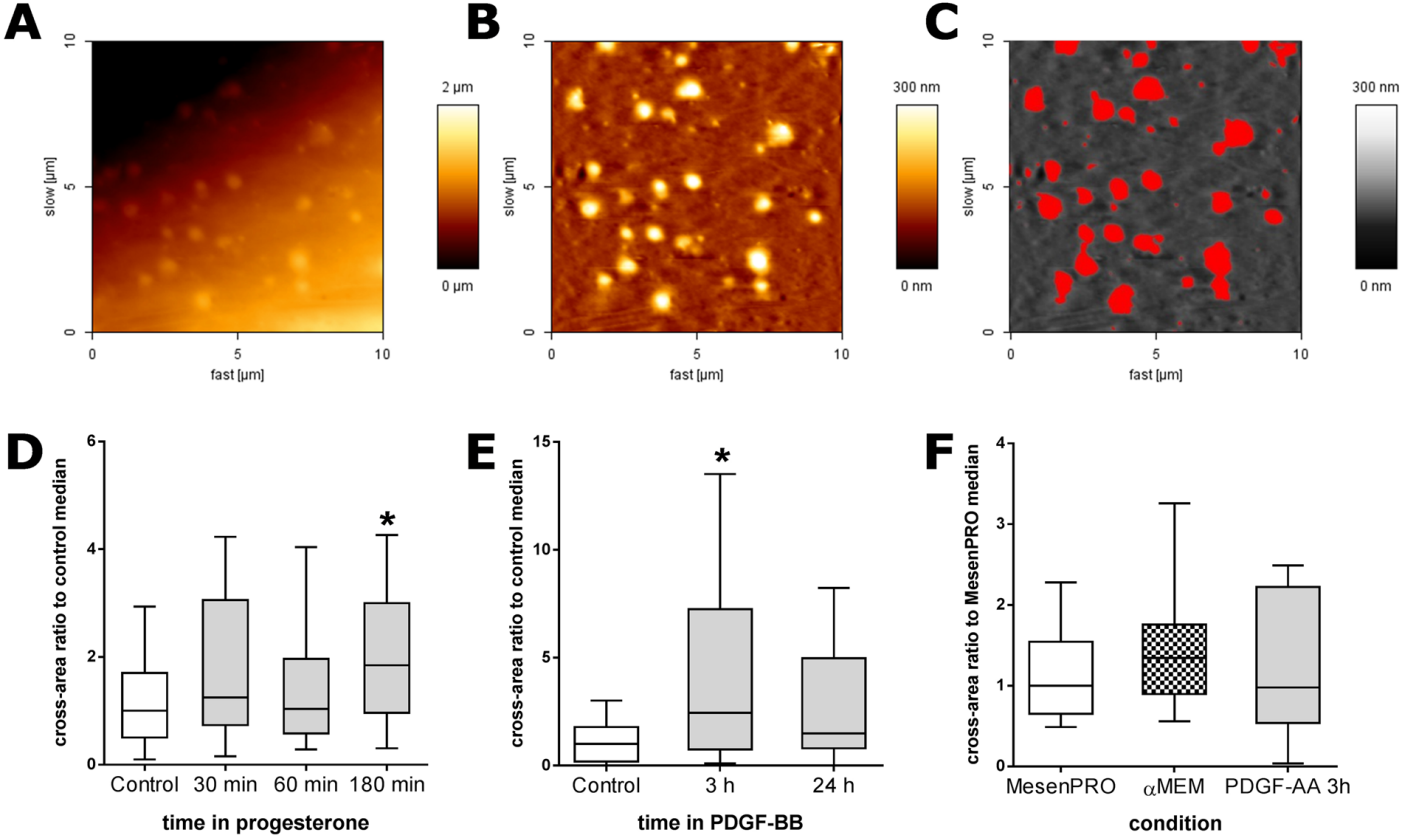
Effect of different treatments or conditions on the amount of membrane protrusions. (**A**) AFM raw topographic image of a 10 × 10 µm area on the surface of an ADSC (this example corresponds to a cell under progesterone treatment for 30 min). (**B**) The same area after flattening, as described in the methods section. (**C**) Cross-sectional areas of protruding elements at 50 nm height above the mean cell surface, in red; these areas were used for quantifying the amount of protrusions in different culture conditions, as shown in (**D**–**F**). (**D**) Early effects of the addition of 0.5 mM progesterone (n = 30). (**E**) Effect of the addition of 10 ng/ml PDGF-BB at 3 h or 24 h (n = 20). (**F**) Effect of culturing on basal medium without serum (αMEM) or of treating the cells with PDGF-AA (n = 10). Tukey’s box and whiskers plots, where the central line represents median values, boxes represent the interquartile range (IQR) and whiskers extend to the extreme values within 1.5 times the IQR. Values have been normalized by dividing by the median of their respective controls (cells maintained in MesenPRO medium in each experiment). In D and E, Kruskal-Wallis one-way analysis of variance showed statistically significant differences (P < 0.05). Asterisks (*) mark the treatments where differences with respect to controls were detected (post-hoc Dunn’s test, P < 0.05).

In average, the amount of protruding cross-area on plasma membranes at 50 nm height over the background could represent from 0.77% to 7% of the membrane surface, as observed by AFM along the different experiments that were performed. Cross-area data distribution did not pass the normality test, due to the weight of single large protrusions compared to those of the more frequent small protrusions. Because of that, non-parametric statistics were used. Statistical analyses of the effect of different treatments or conditions on the amount of protruding bulges on the ADSC surface were performed by AFM on fixed cells (Fig. 5). Measurements of the total cross-area at 50 nm height in 10 × 10 µm fields of the cell surface showed that 0.5 mM progesterone or 10 ng/ml PDGF-BB produced statistically significant increases in the amount of protruding cross-area at 3 h with respect to untreated cells (Fig. 5D,E). Maintenance in a defined medium without serum (medium α-MEM) or treatment with 10 ng/ml PDGF-AA did not produce statistically significant differences with respect to maintaining cells in the ADSC growth medium (MesenPRO) (Fig. 5F).

## Discussion

The EVs released by MSCs are a promising material for future cell-free bio-therapeutic approaches in organ regeneration and immune modulation^6, 25, 26^. The population of therapeutically active EVs is formed by exosomes and shedding vesicles (also known as microparticles, microvesicles or ectosomes), but the contribution of each of these two subpopulations to the EV pool and to their activities has not been identified yet. Indeed, they are generated through two very different cellular mechanisms: while the exosomes originate in the late endosomal compartment, the shedding vesicles stem as plasma membrane protrusions. Their molecular content is, therefore, different and so will likely be their action on target cells. Identification of exosomes and shedding vesicles in the EV pool often relies on the presence of molecular markers of the endolysosomal compartment on their membrane, on their size, or on their isolation through differential ultracentrifugation, but no method has been able yet to segregate these two types of secreted vesicles^31^. Both in the literature and in commercially available isolation products, the generic term “exosome” is often used to refer to small EVs, which creates confusion^32^ since *bona fide* exosomes and shedding vesicles may act differently on target cells^33^.

We are adding here new knowledge about the dynamics of plasma membrane protrusions, which are the likely origin of shedding vesicles. To that goal, we have used AFM measurements in living cells as well as AFM and SEM in fixed cells. The first conclusion is that protrusions (and some pits) are being continuously formed on the plasma membrane and are not a fixation artifact. Recordings of the cell surface topography showed that protrusions emerge and disappear in 10–20 min. The presence of pits, which could be linked with openings of multivesicular bodies to the plasma membrane to release exosomes, could not be unambiguously identified in living cells, although long-lasting larger pits were observed.

The surface of most ADSCs exhibit hundreds of protruding bulges. These are mostly round membrane protuberances that commonly stand out 20–300 nm from the surrounding surface. They differ from other reported membrane structures observed by AFM^34–36^ because they are smaller and they can be seen to detach *in vivo*. We have previously calculated that, in our culture conditions, ADSCs may release more than 30,000 EVs per cell in 24 h and that progesterone even increases those numbers^37^. *In vivo* AFM here presented shows a fast turnover of membrane protrusions, each lasting less than 20 min. Using those numbers we may estimate the production of tenths of thousands protrusions by each cell per 24 h, which is in the range of the number of EVs that we have counted. Detailed quantification needs to be undertaken but our initial data suggest that shed vesicles may account for, at least, comparable numbers to exosomes in the EV pool.

Long term recording of living cells was subjected to the natural cell movements as well as to morphological changes of the cell surface, some of which might be in response to a mechanical stimulation of the AFM probe. In spite of this, 1–2 µm displacements of protrusions or pits during recording, with respect to neighbouring elements, were observed. On the other hand, during AFM phase mode characterization on some ADSCs, we found large obscured regions extending further from the cell body (see, for instance, Fig. 1A). This contrast is commonly related to stiffness variations on the surface^38^ and may represent areas of accumulation of extracellular matrix (like hyaluronic acid), not allowing the measurement of membrane protrusions underneath. The interaction of extracellular matrix components with the free circulation, release or attachment of EVs merits further studies. Since those gelatinous-type of regions are not observable in SEM or TEM, the use of AFM would be of interest for such studies.

Height of protrusions with respect to the surrounding surface is the AFM measurement that better reflects the actual size of bulges, since lateral measurements would be affected by the probe tip shape and size. Most of the protrusions raised less than 100 nm, so it is likely that most vesicles that are shed from ADSCs will have that diameter or smaller. These measurements, obtained by AFM on the cell surfaces of living cells, were supported by AFM measurements, as well as by SEM images, performed in fixed cells. Our data suggest that a large population of the EVs of less than 100 nm originate as shedding vesicles and, thus, overlap largely with the exosome sub-population. So, the distinction of exosomes and shedding vesicles by differential centrifugation, which is essentially a size-based technique to discriminate EVs, appears to be less than optimal.

In the present report, we have tried to provide measurements that would relate to the amount of protrusions so comparisons of the effects of different treatments or culture conditions could be evaluated by AFM. We used the total cross-area that was measured at 50 nm heights in randomly chosen 10 × 10 µm membrane fields as a possible estimate of the amount of potential vesicles that would be shed. We chose the 50 nm because this was the minimum height at which most circular protrusions were consistently discriminated from other irregularities of the cell surface (see Fig. 5C), while above that height many protrusions were missed (not shown). The cross-area values obtained from cells in control conditions did not pass D’Agostino & Pearson omnibus normality test, for which non-parametric statistics were used to compare different conditions.

As a test of this semi-quantitative procedure, ADSCs were treated with drugs that were known to stimulate the number EV secretion. We had previously observed^37^ that the addition of 0.5 mM progesterone to the culture medium increased the number of EVs collected in 24 h, as measured with Nanoparticle Tracking Analysis or counted with AFM after seeding onto poly-L-ornithine coated coverslips. Additionally, Lopatina *et al*.^39^ had reported that PDGF (actually, PDGF-BB, personal communication) stimulates the secretion of EVs in ADSCs. We thus used these two treatments to evaluate if total cross-area measurements at 50 nm height increase similarly. Indeed, such measurements were in agreement with a stimulation of EV production in ADSCs by progesterone or PDGF-BB. Increased amount of protrusions was evident already at 30 min after progesterone addition and the difference was statistically significant by 3 h. The effect of PDGF-BB was tested at 3 and 24 h after its addition to the culture medium. As mentioned above, a previous report^39^ showed that EVs numbers were increased in the 24 h-conditioned medium under PDGF-BB treatment and suggested that such increase could be attributable to the secretion of vesicles of smaller size. Here we found that by 3 h there was a statistically significant increase in the cross-area of protrusions at 50 nm height. However, by 24 h that difference had disappeared. Our findings suggest that, at least in relation to the contribution of shedding vesicles to the EV pool, most of the stimulated release occurs during the first 24 h. Other conditions, like culturing in serum-less medium (α-MEM) or treating the cells with PDGF-AA did not produce changes in the amount of protrusions, as estimated by our procedure. There are, however, some limitations to the above procedure. Scanned areas did not include perinuclear regions, cell edges or filamentous processes, which were often decorated by numerous bulges, as shown in Fig. 4B. So, the present measurements should be taken only as a first approach to estimate the timing at which different treatments may have effect on the formation of membrane protrusions.

The observed pits in the ADSC surface could be interpreted as exosome-releasing MVBs at the moment of fusing to the plasma membrane and opening to the extracellular space. When recorded by AFM in living cells, only large pits were unambiguously identified, while depressions of smaller diameter could not be confidently recognized. Large pits (0.4–2 µm wide) remained opened for few minutes to more than 1 h and might move laterally short distances by the membrane. In fixed cells, the size of identifiable pits was smaller. It might be due to a fixation artifact but their size (commonly 200 nm) is coherent with what is usually depicted for MBVs by transmission electron microscopy. Indeed, the presence of spherical profiles of CD63 immunofluorescence underneath some pit clusters (see Supplementary Information Fig. 2) might suggest their relationship with multivesicular bodies.

Alternatively, the observed membrane depressions might correspond to caveolae or clathrin-coated pits, two types of lipid rafts invaginations involved in endocytosis. The size of caveolae openings to the surface (around 45 nm in diameter^40^) is smaller than that of the pits that we can confidently show here *in vivo*. Additionally, we have not observed immunofluorescent staining for caveolin-1 clustered at the pits recorded by AFM (see Supplementary Information Fig. 3). So, the pits that we report here do not appear to be caveolae. Clathrin-mediated endocytosis and macropynocytosis is involved in exosome uptake^41^ and the size of clathrin-coated pits is similar to the pits here imaged by AFM. Superresolution fluorescence microscopy for clathrin in combination with AFM would be required to ascertain if any of these pits are involved in endocytic or pynocytic processes. Some large pits might represent porosome complexes^42^, which are groups of membrane depressions where secretion of effector molecules takes place in endocrine, exocrine and neural cells. However, the inner surface of pits of the porosome complex can be readily measured by AFM^42^ because they are shallow depressions, while the pits here shown exceed the probe tip capacity to measure their depth.

It is interesting to note, on the other hand, that we did not find evidence that treatment with progesterone or PDGF-BB increased the number of identifiable pits of plasma membranes although this subject merits a detailed quantification using different techniques to ascertain if the increase of extracellular vesicle content in the medium by these factors combines both exosome and shedding vesicle release or if it is essentially due to this last process, as our observations might suggest.

## Conclusions

Adipose tissue-derived MSCs display large number of bulges and variable number of pits on their surface, as shown by AFM and SEM. The bulges had similar size distribution as the extracellular vesicles laid on the culture surface, so they appear to be the origin of the shedding vesicles. AFM allows measuring the dynamics of generation of these bulges in living cells. Cross-area measurements at 50 nm height over the cell surface provided values that matched the increased EV release by ADSC when stimulated with progesterone or PDGF-BB. The present results suggest us that shedding vesicles may constitute a relevant population of the EVs released by stimulated adipose-derived MSCs.

## Methods

### Cell cultures

Human ADSC cultures were obtained from 0.5–1 g pieces of subcutaneous inguinal fat that was discarded during the course of vascular surgeries in patients of either sex with no underlying pathology and ages from 29 to 72 years. All procedures were performed in accordance to Spanish regulations (Law 14/2007 and Royal Decree 1716/2011). The samples were obtained after the patient’s informed, written consent following protocols that were approved by the Ethics and Clinical Research Committee of the Hospital Universitario Ramón y Cajal.

The pieces of adipose tissues were placed in Leibowitz’ L-15 with penicillin + streptomycin + amphotericin B (antibiotic/antimycotic by Gibco-Life Technologies) and kept at 4 °C for up to 4 h until processed. After detaching major fasciae and blood vessels under a dissecting microscope in sterile conditions, the fat tissue was trimmed into small pieces. These were then digested with collagenase A (Sigma-Aldrich), 1 mg/ml, in MesenPRO medium (Gibco/Invitrogen) for 35–40 min at 37 °C. Subsequently, the pieces were transferred to a tube with fresh maintenance medium and mechanically dispersed by repeated pipetting using a P1000 automatic pipette with fire-smoothened filtered sterile tips (Sorenson). The cell suspension was seeded on a 75 cm^2^ culture flask (Falcon) in MesenPRO medium. After 24 h, the medium was withdrawn and the flask was rinsed twice with Hank’s balanced salt solution (HBSS without Ca^2+^ and Mg^2+^, Sigma-Aldrich), and fresh MesenPRO was added. The medium was changed the 4th day and passages were performed every 7 days. Passages were done by digesting near-confluent cultures with trypsin 0.05% + EDTA 0.02% (Sigma-Aldrich) in HBSS for 10 min at 37 °C and then seeding dispersed cells at 660 cells/cm^2^ in new flasks. The present studies were performed on ADSCs at passages 2–7, during which cells multiplied 30–50 times per week.

Cells used for experimental procedures were maintained in vesicle-depleted MesenPRO. MesenPRO RS™ Medium has been specifically formulated by Gibco to support the growth of human MSCs in culture. It is made of MesenPRO basal medium added with L-glutamine and MesenPRO-supplement (2%), which contains undisclosed components and serum. To prevent contamination of experiments with possible EVs carried by that serum, MesenPRO supplement was ultracentrifuged at 100,000 × *g* for 70 min at 4 °C and supernatants aliquoted and frozen at −80 °C for its use as needed for making MesenPRO medium. No difference was observed between adipose-derived MSCs grown in regular MesenPRO medium and those grown in centrifuged MesenPRO. Nevertheless, as shown in the Supplementary Information, neither vesicle-depleted MesenPRO medium nor regular unprocessed MesenPRO medium contain measurable amounts of EVs. This contrasts with fetal bovine serum-containing medium, which carries large amounts of EVs that become deposited on the culture surface (Supplementary Information Fig. 1).

### Atomic force microsocopy

AFM was performed in a JPK NanoWizard II® AFM coupled to a Nikon Eclipse Ti-U inverted optical microscope. Dynamic mode immersed in liquid with an Olympus commercial silicon nitride cantilever tip (0.76 N/m, 71 kHz), with typical 15 nm radius at the end, was used. *In vivo* analyses were performed at 37 °C constant temperature maintained by a JPK BioCell thermocontroller. AFM characterizations on fixed cells were done after adding equivalent amounts of paraformaldehyde 4% to the medium for 10 min and changing to glutaraldehyde 2.5% for 2–3 h. For the study of the effect of drugs on the generation of cell protrusions, 0.5 mM progesterone (water soluble, Sigma-Aldrich), 10 ng/ml PDGF-BB or 10 ng/ml PDGF-AA (both from ImmnoTools GmbH) were added to the MesenPRO medium that had been changed 1 h before so that pH and temperature were stabilized at the moment of the treatment. Experimental conditions also included incubation in defined medium (α-MEM, Sigma-Aldrich) that had been pre-warmed and pre-equilibrated for pH. The cells were fixed, as above, at different times after the addition of drugs or medium change.

### Scanning electron microscopy (SEM)

Series of ADSCs grown on glass coverslips were fixed with 2% paraformaldehyde in 100 mM cacodylate buffer for 10 minutes followed by a stronger fixation with 2% paraformaldehyde + 2,5% glutaraldehyde + 2% tannic acid in 100 mM cacodylate buffer for 1 hour at 4 °C. Then, the samples were stored in 0,01% buffered glutaraldehyde until finally processed. For SEM analysis, cells were extensively washed with cacodylate buffer, post-fixed with 1% OsO_4_, washed again with buffer and dehydrated using graded ethanol series and acetone, completely dried (critical-point method) and metal-coated with gold/palladium alloy.

### Measurement of protrusions by AFM

To estimate the size of protruding bulges on the cell membranes, 10 × 10 µm fields on the surface of different ADSCs were randomly selected and scanned by AFM. Images were flattened by 9^th^ grade polynomial fit subtraction on each scan line independently, using JPK Data Processing software. In this way, cell surface slopes not related to protrusions can be filtered in the analysis. Using Wasabi! software, cross-area above a fixed height threshold was measured on each image, in order to produce a value representing the amount of protrusions in that field.

### Statistical analyses

Data were analyzed with the help of GraphPad Prism (v. 6.01). Non-parametric statistics were used. Cross-area measurements of each experiment were normalized by dividing them by the median of control groups due to the inter-experiment variability. Data were represented as Tukey’s box and whiskers plot. Data were compared by using Kruskal-Wallis one-way analysis of variance followed by Dunn’s multiple comparisons test. Differences were considered statistically significant when P ≤ 0.05.

## Electronic supplementary material

Supplementary PDF File
