# Supplementary material for: Dynamics of plasma membrane surface related to the release of extracellular vesicles by mesenchymal stem cells in culture
Source: Sci Rep. 2017 Jul 28;7:6767. doi: 10.1038/s41598-017-07265-x (PMC5533874; doi:10.1038/s41598-017-07265-x)
Supplement: Supplementary file 1 — Supplementary PDF File [file 41598_2017_7265_MOESM1_ESM.pdf]

__Supplementary information__

Dynamics of plasma membrane surface related to the release of extracellular vesicles by mesenchymal stem cells in culture

Santiago Casado, Maria del Val Toledo Lobo and Carlos Luis Paíno

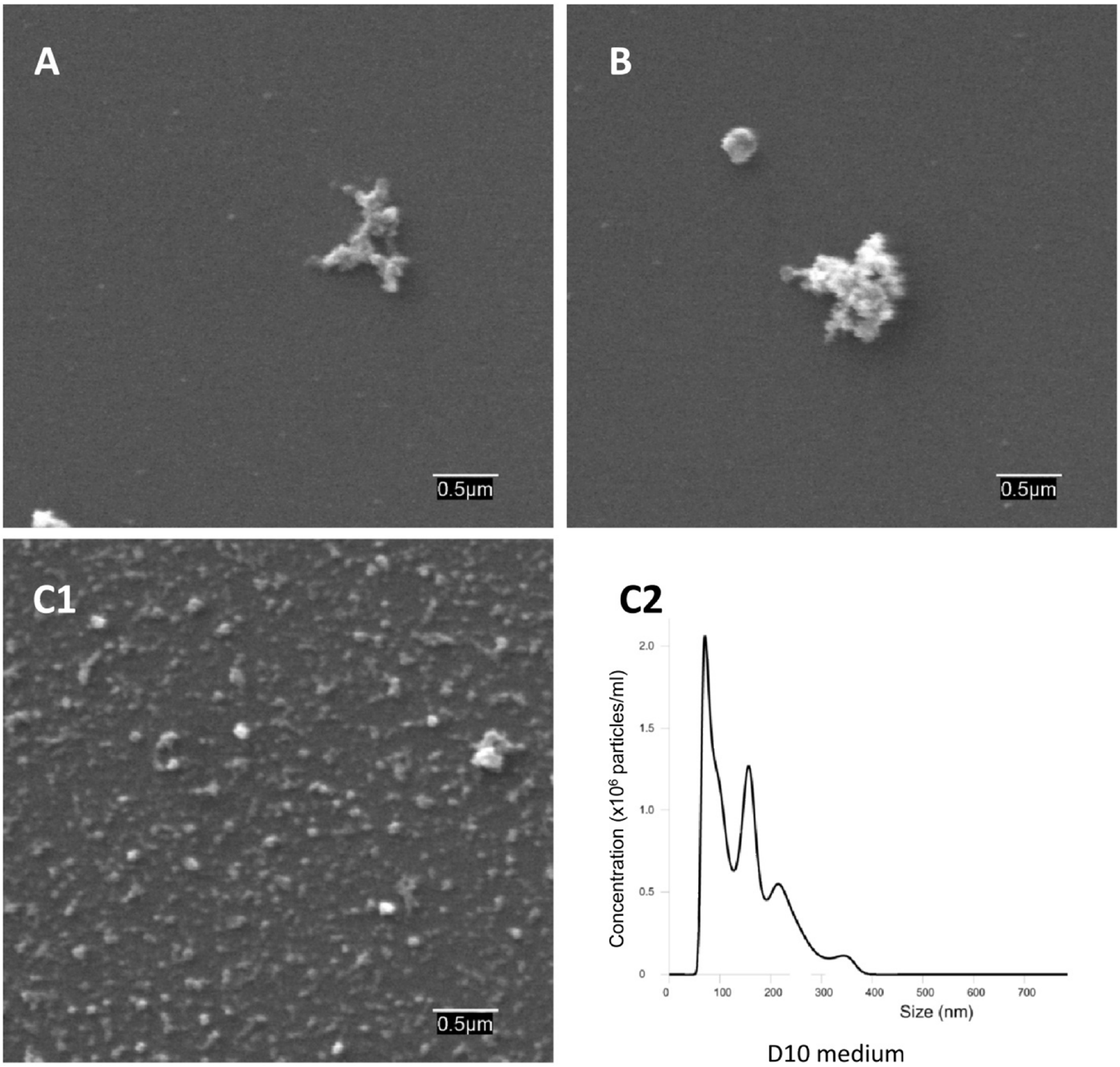

S.I. Fig 1. Checking of extracellular vesicles in culture media. Cell-free coverslips were incubated for 48 h at 37 ºC with vesicle–depleted MesenPRO medium (**A**), regular, unprocessed MesenPRO medium (**B**) or Dulbecco's Minimal Essential Medium added with 10% qualified Fetal Bovine Serum from Gibco, here named D10, (**C1**), for comparison. Scanning electron microscopy showed that neither vesicle-depleted MesenPRO nor unprocessed MesenPRO laid EVs on the coverslip surface and only occasional unidentified clumps (shown here as reference point for focusing) were observed. By contrast, incubations in D10 medium showed large amounts of EVs deposited on the coverslip surface. Analysis of the same media by Nanoparticle Tracking Analysis (NanoSight) proved that no nanoparticle was detectable in either vesicle-depleted MesenPRO or in regular untreated MesenPRO (not shown), whereas D10 contained numerous EVs (as shown in **C2**).

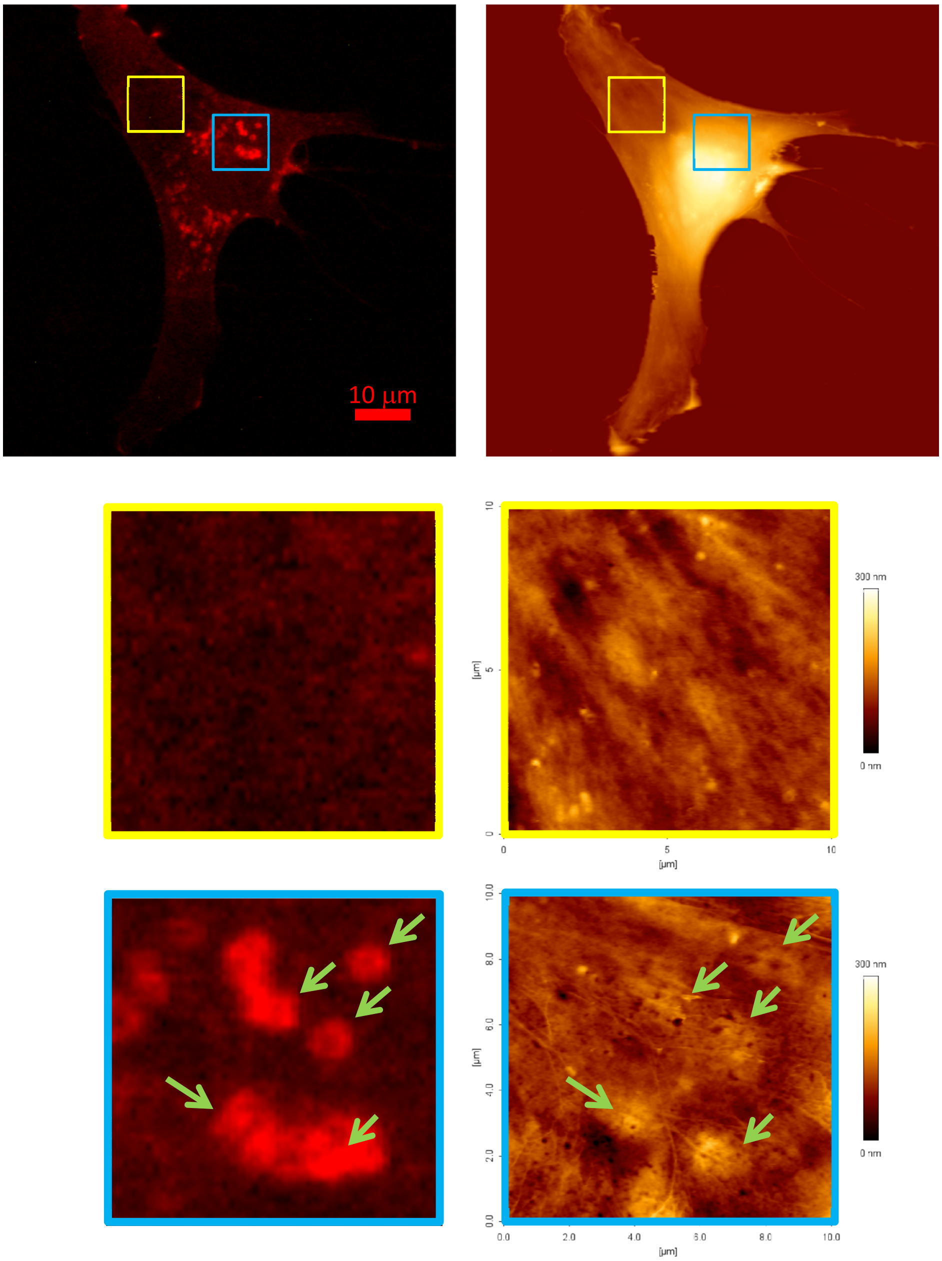

S.I. Fig 2. Combination of immunofluorescent staining for CD63 (left) and AFM (right) in the same cell. The two lower rows are 10 x 10 µm details of the areas framed with yellow and blue squares on the cell. It can be observed that, in some instances, small pits are clustered at places where strong, spherical immunostaining of CD63 is visible (green arrows), although it cannot be resolved whether they co-localize or they are located at different depths.

Methods: Cells were initially fixed with 4 % paraformaldehyde + 0.1 % glutaraldehyde in PBS. Blocking solution for immunostaining was made with 5% normal goat serum + 0.1 % saponin. Coverslips were reacted overnight with mouse anti-CD63/LAMP-3 (DSHB, clone H5C6), diluted 1:100 in blocking solution. Afterwards, the coverslips were incubated in the dark for 30 min with Alexa 568-conjugated anti-mouse IgG (H+L), diluted 1:500 in blocking solution. The cultures where then post-fixed in 2 % paraformaldehyde + 2.5 % glutaraldehyde for 30 min and kept in PBS + 1% of the fixative in the fridge until microscopy. Coverslips were positioned in the inverted microscope (see Methods in the main text) and cells were visualized through a 40X dry objective (PlanFluor, NA: 0.75).

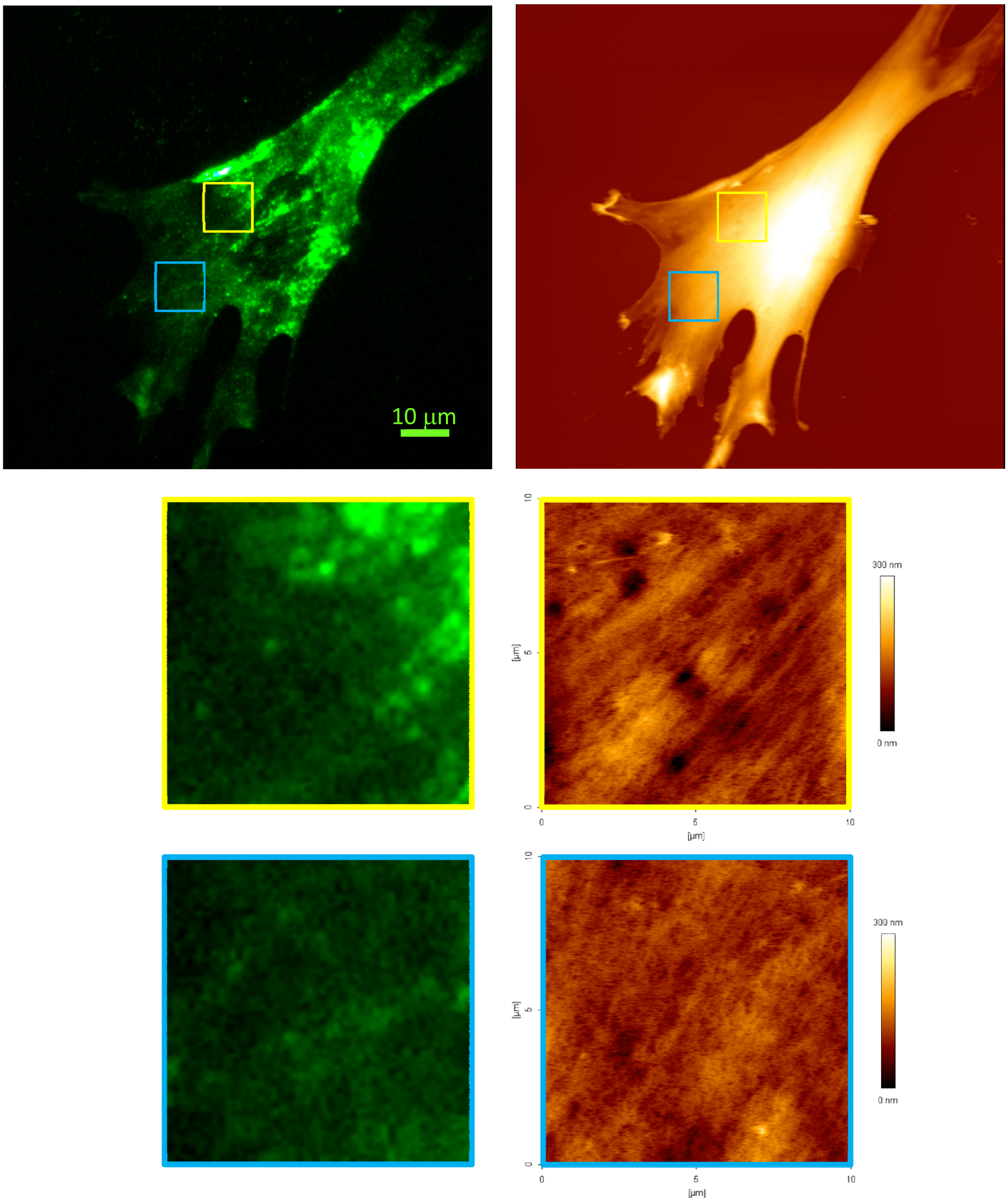

S.I. Fig 3. Combination of immunofluorescent staining for caveolin-1 (left) and AFM (right) in the same cell. The two lower rows are 10 x 10 µm details of the areas framed with yellow and blue squares on the cell. It can be observed that pits do no keep relationship to immunofluorescence intensity (however, note that the resolving power of regular fluorescence microscopy is much poorer than that of AFM and does not allow co-localization)

<u>Methods</u>: Cells were initially fixed with 4 % paraformaldehyde + 0.1 % glutaraldehyde in PBS. Blocking solution for immunostaining was made with 5% normal goat serum + 0.1 % saponin. Coverslips were reacted overnight with rabbit anti-caveolin 1 (Santa Cruz sc-894), diluted 1:100 in blocking solution. Afterwards, the coverslips were incubated in the dark for 30 min with Alexa 568-conjugated anti-mouse IgG (H+L), diluted 1:500 in blocking solution. The cultures where then post-fixed in 2 % paraformaldehyde + 2.5 % glutaraldehyde for 30 min and kept in PBS + 1% of the fixative in the fridge until microscopy. Coverslips were positioned in the inverted microscope (see Methods in the main text) and cells were visualized through a 40X dry objective (PlanFluor, NA: 0.75).
